# Supplementary material for: Application of Maillard Reaction Products Derived Only from Enzymatically Hydrolyzed Sesame Meal to Enhance the Flavor and Oxidative Stability of Sesame Oil
Source: Molecules. 2022 Dec 13;27(24):8857. doi: 10.3390/molecules27248857 (PMC9781902; doi:10.3390/molecules27248857)
Supplement: Supplementary file 1 [file molecules-27-08857-s001.zip › molecules-2070932-supplementary.pdf]

Electronic supporting information

Application of Maillard reaction products derived only from enzymatically hydrolyzed sesame meal to enhance the flavor and oxidative stability of sesame oil

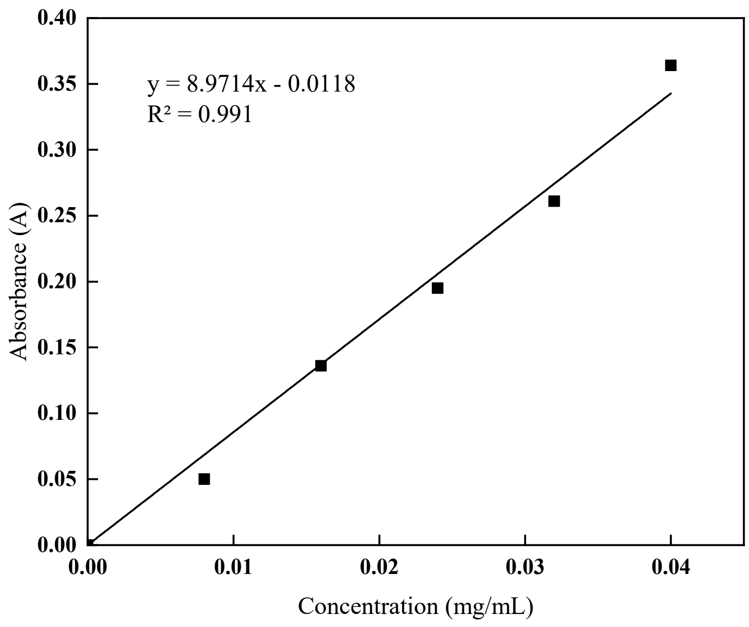

Figure S1. Standard curve of reducing sugar content.

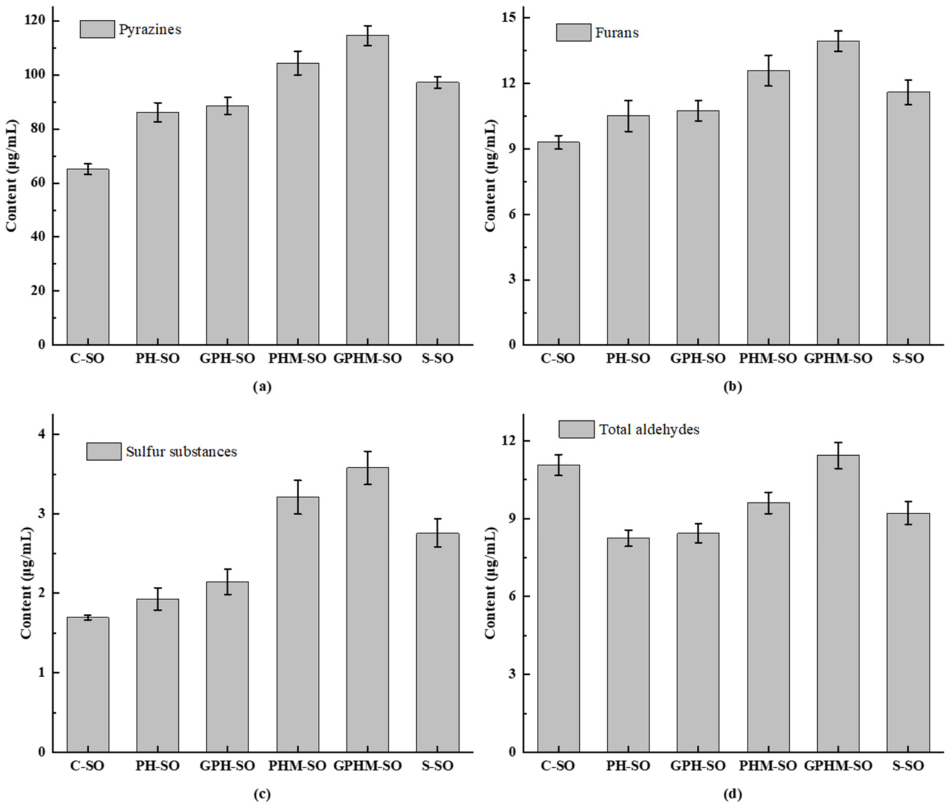

Figure S2. Four main flavor substances contents in different oils.

(a) Pyrazines, (b) furans, (c) sulfur substances, and (d) aldehydes.

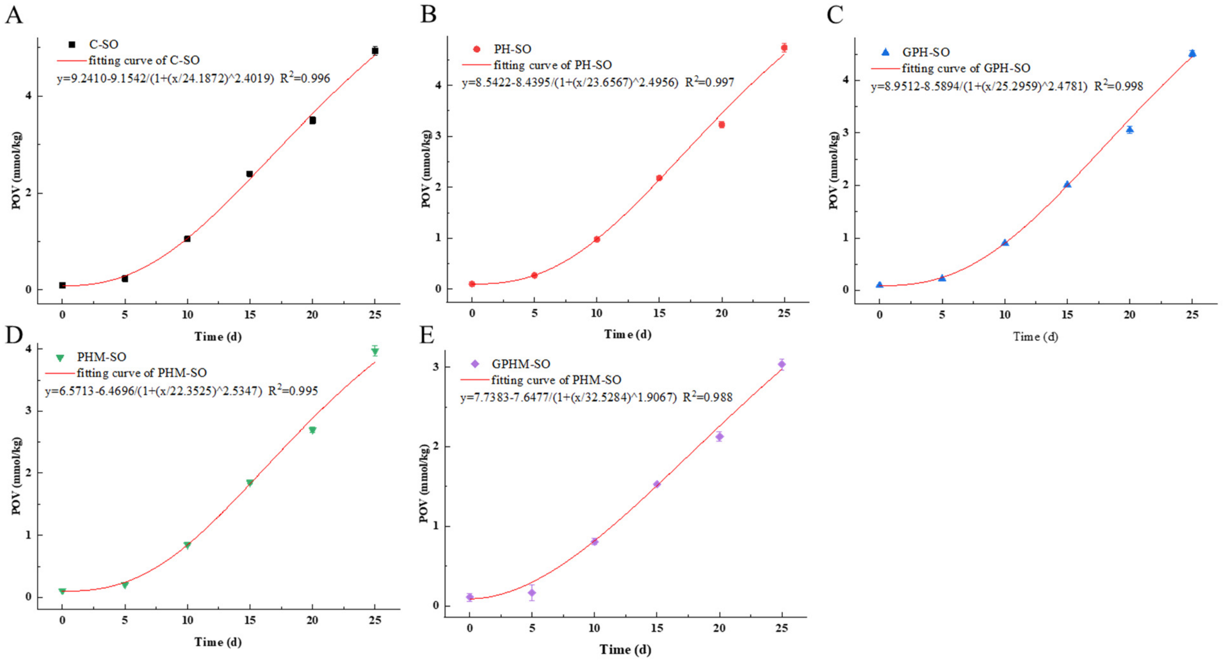

Figure S3. Fitting curves between peroxide value (y) and storage time (x) of different oil samples in accelerated oxidation process.

Fitting curve for C-SO was (a), for PH-SO was (B), for PH-SO was (C), for PHM-SO was (D), and for GPHM-SO was (E).

Table S1. The sesame oil fragrance descriptors definition.

Sensory Indicators	Judging controls	Scoring Criteria
**Toasted sesame aroma**	The best standard is the aroma of raw sesame roasted at 150 °C for 10 min.	Strong odor: 7-10 points; Medium odor: 5-6 points; Lighter odor: 2-4 points; Odorless :0-2 points.
**Caramel aroma**	Based on caramel aroma baked at 170 °C for 5 min.	Strong odor: 7-10 points; Medium odor: 5-6 points; Lighter odor: 2-4 points; Odorless: 0-2 points.
**Aroma persistence**	Based on the primary sesame oil sold on the market, counted as 5 points.	Strong persistence: 7-10 points; Medium persistence: 5-6 points; Lighter persistence: 2-4 points; Less persistence: 0-2 points.
**Off-flavor**	The standard is the aroma of raw sesame roasted at 250 °C for 10 min.	Odorless: 0-2 points; Lighter odor: 2-4 points; Medium odor: 5-6 points; Strong odor: 7-10 points.
**Overall satisfaction**	**The evaluation based on toasted sesame aroma, caramel aroma, aroma persistence and off-flavor.**	

Note: the score was given on a scale of 0 (undetected) to 10 (strong) for the flavors of toasted sesame aroma, caramel aroma and aroma persistence; the score was given on a scale of 10 (strong) to 0 (undetected) for off-flavor.
